# Inflammation of the Iris

**Published:** 1910-10-15

**Authors:** A. Maitland Ramsay

**Affiliations:** Ophthalmic Surgeon, Glasgow Royal Infirmary, and Lecturer on Eye Diseases, Queen Margaret College, University of Glasgow.


### Hospital Clinics.

### INFLAMMATION OF THE IRIS.

By A. MAITLAND RAMSAY, M.D., F.R.F.P.S.G., Ophthalmic Surgeon, Glasgow Royal Infirmary, and Lecturer on Eye Diseases, Queen Margaret College, University of Glasgow.

(A Clinical Lecture delivered in the Glasgow Ophthalmic Institution on June 16, 1910.)

(*Continued from page* 42.)

The third patient is a woman of twenty-two years of age, who was also admitted to the institution on June 11, 1910. She, too, is suffering from iritis, but the onset and course of the symptoms are in marked contrast to those just described in connection with our second patient. In the case of this girl the history of eye trouble begins only three and a half months before her admission to the hospital, at which time the right eye became affected. The onset was most insidious: she had been "run down" in health, and, as she says, "caught cold in her eye," which then became injected and watered readily when the light was bright. She made no complaint of pain, and was always fit for her work as domestic servant; but the eye was never quite free from redness, and the lids always tended to close when she faced the light, There was also times when, owing to her having, as she thought, caught fresh cold, the eye became redder than usual, the intolerance of light amounted almost to pain, and the lachrymation was constant and copious. A fortnight before she was admitted to the institution the eye became much more troublesome, and she noticed, for the first time, that vision with it had got very indistinct. There was a feeling that everything she looked at was misty, and when she closed the left eye she found herself quite unable to read. From this time matters grew steadily worse, and though there was no acute pain, there was constant aching, especially at night.

### A Syphilitic or Tubercular Case.

The right eye is, as can be easily seen, red; and for the most part the injection is pink, deep-seated, and most intense around the cornea; the anterior chamber is deep, the aqueous is muddy, and there are numerous spots of exudation adherent to the posterior surface of the cornea; the iris is infiltrated, much discoloured, and bound down to the lens capsule, while posterior synechiæ extend so firmly round the pupillary margin that atropine dilates the pupil only at its upper aspect, the inner, outer, and lower boundaries remaining fixed. On the anterior surface of the iris, below and slightly to the nasal side, is a nodule, orange-yellow in colour, and rather larger than a pinhead. No blood vessels are visible over its surface, but the capillaries are greatly injected all round its base. A similar but much smaller nodule occupies the corneo-iridic angle below, and to the outer aspect of, the larger nodule. The intraocular tension is much reduced, there is no tenderness on pressure over the ciliary region, the fundus cannot be illuminated, and sight is reduced to a perception of hand movements. The left eye appears to be normal both in structure and in function.

In this case the orange-yellow nodules are the most characteristic objective features, and at once suggest that the predisposing cause is either syphilis or tubercle. There is nothing in either the personal or the family history of the patient to support the former view; but the latter theory is confirmed both by the course of the inflammation and by the results of inoculation by tuberculin. It is true that Moro's tuberculin ointment test gave a negative result, but the subcutaneous injection of a minute quantity of Koch's Old Tuberculin was followed in a few hours not only by a rise in temperature to 100°.2 Fahr., but also by marked reaction in the eye itself. There can, I think, therefore, be no doubt that we have here a case of tuberculous iritis, and one naturally asks whether the ocular manifestation be primary or secondary. The most careful examination has failed to reveal the existence of tubercle in the chest, in the abdomen, or in any other part of the body, nevertheless the probabilities are that there is somewhere a hidden centre of disease, from which the infection has reached the eye. It is, indeed, very doubtful if primary tuberculosis of the eyeball ever occurs. Its appearance there ought rather to be regarded as a local manifestation of a general infection of the blood; and in many instances the eye symptoms mark the beginning of a series of successive local infections, which may terminate in *phthisis pulmonalis.*

### Tubercular Iritis.

Tubercle is one of the rarest predisposing causes of iritis, but the experience of recent years leads me to think that perhaps ocular tuberculosis is of more frequent occurrence than is commonly imagined. In this patient the infection seems to be attenuated, and the prognosis is on the whole favourable. Indeed, the prognosis of tuberculous iritis is always more favourable in the adult than in the child, because in the latter the eye may speedily burst, and death ensue from meningitis. The treatment in the case before us will consist, in addition to the usual local remedies, of regularly repeated injections of tuberculin, supplemented by the internal administration of quinine and grey powder alternating with the perchlorides of iron and mercury. The value of small doses of perchloride of mercury in chronic tuberculous disease was well known in the old clinical practice, and ought not to be forgotten. The girl will also be kept as much in the open air as possible, and the diet will be carefully attended to.

### The Fourth Case.

The fourth patient is a woman of thirty-six years of age, who was admitted to the institution on June 14, 1910. Four weeks before that date her left eye began to water freely on exposure to light, and became painful, the pain, which radiated from the eye along all the branches of the fifth cranial nerve, growing steadily worse as time went on. It ultimately became so severe at night that she could not sleep, and this drove her to the hospital for relief. At the time of her admission the left eye showed intense pericorneal injection; the iris seemed to be thickened, and its surface had lost its natural brilliancy owing to being covered by a layer of inflammatory exudation, some particles of which had fallen into the aqueous and rendered it muddy, while others had adhered to the posterior surface of the cornea, and the pupil was contracted and irregular in outline, and when it was placed under the influence of atropine, posterior synechiæ numerous and firm, were readily seen. The intra-ocular tension was normal, there was tenderness on pressure over the ciliary region, the fundus could not be illuminated, and vision was reduced to a perception of hand movements.

### The Signs.

There is nothing in the objective appearances to give a clue to the ætiology of the iritic inflammation, but the personal history affords a strong presumption that the predisposing cause is syphilis. Eight years ago the patient had a miscarriage at the third month, and three months before her admission she had another miscarriage also at the third month. In the intervening period she has borne six children, all of whom are alive and healthy. Two and a half years ago she suffered from extra-uterine gestation. She says that for the last month or six weeks her hair has been falling out, while careful examination reveals some copper-coloured spots on her arms and chest. There are also superficial erosions on the side of the tongue near the tip, a mucous patch on the buccal mucous membrane close to the left angle of the mouth, and marked injection of both tonsils, the right being ulcerated. The aqueous humour was drawn off and examined, but the *spirochæte pallida* was not found. An examination of the blood, however, which gave a markedly positive Wassermann reaction, converted the suspicions roused by the personal history into a practical certainty, so that there can, I think, be no doubt that here we have a case of syphilitic iritis.

### Syphilitic Iritis.

It is usually said that inflammation of the iris of luetic origin presents well-marked signs and symptoms, most prominent among which are turbidity of the aqueous, greasy spots on the posterior surface of the cornea, opacities in the vitreous, a degree of dusky red pericorneal injection out of all proportion to the severity of the pain (for the most part circum-orbital and nocturnal), and, last and most important, the formation in the iris of nodules generally situated close to the margin of the pupil, although they may also occur at the ciliary attachment as well as in the substance of the iris itself. These nodules vary in size, are usually orange-red in colour, and may not be visible till after the dilatation of the pupil by atropine, when they can be detected on the parts adherent to the anterior capsule of the lens.

### The Treatment of Luetic Iritis.

Of all the causes of iritis, syphilis is the most frequent, the inflammation in such circumstances often involving both eyes. When this is so, the one is, as a rule, affected after the other, but it is by no means unusual to find both suffering at the same time. Relapses in the form of frequently recurring attacks are not uncommon, but iritis due to syphilis is liable to be complicated with, or to be followed by, inflammation of the choroid and of the retina; and hence, while the usual local treatment must be carried out thoroughly and efficiently, an equally vigorous constitutional anti-syphilitic treatment must be simultaneously instituted in order to prevent the complications and sequelæ which are otherwise almost certain to occur. The ocular tissues are so delicate that they are quickly damaged, and sight may, with equal quickness, be permanently impaired. It is, therefore, most important to act with promptness and energy, so that the patient may be brought under the influence of mercury with the least possible delay. I say under the influence of mercury, and in saying so I am not unmindful of the value of serum therapy in syphilis, nor do I forget the eulogy that has been bestowed on atoxyl, soamin, and other arsenical compounds as substitutes for mercurial preparations.

Valuable as these means are, our chief reliance in syphilitic cases must still be placed on mercury. What should be aimed at is to saturate the tissues as rapidly as possible, and at the same time to secure steady and gradual elimination of the drug from the system. Every patient must, of course, be dealt with according to the special indications presented by his particular case, but as a general rule calomel and opium in pill form is the best routine treatment. The opium serves the double purpose of relieving pain and of locking the calomel in the system, so that the constitutional effects of the latter are speedily manifested. One ordinary pill (2 grains of calomel and 1 grain of opium) is administered every night at bedtime, and usually in from four to eight days the patient begins to show signs of salivation. On the appearance of soreness of the gums and increased flow of saliva the acute inflammatory eye symptoms begin to abate, and posterior synechiæ, which up to this point have resisted the action of atropine, now give way and the pupil dilates. It occasionally happens that the pills cause an intense feeling of malaise, with sickness and vomiting, and in these circumstances they must be discontinued, and the mercury administered by inunction. In urgent and difficult cases, indeed, the latter method of treatment should supplement the administration of the drug by the mouth, and by this double means rapid and satisfactory results are often obtained. Not only, however, must the patient be brought quickly to the border of salivation, but he must also be kept under the influence of mercury for weeks, and even for months, after the eye shows no sign of inflammation. All this must, besides, be accomplished without inducing active salivation: the patient must never be salivated. It is all-important, too, that he keep his teeth clean, and for this purpose he should use a chlorate of potash mouth-wash freely.

Mercury and Iodides.

If care be taken to regulate the dose properly, the beneficial action of the drug can be readily maintained without any injurious influence. The pills must be administered at varying intervals according to the constitutional effects which they produce—every second night, or every third night, or once a week, as the case may be. After a course of these pills the preparation of mercury may be changed, the calomel being discontinued and perchloride substituted. At this stage also the iodides will render important service, and I feel sure that their therapeutic efficacy is increased if they are combined with sarsaparilla, while iodism is in great measure prevented if one or two drachms of pepsencia be added to each dose.

The iodides of potassium, sodium, and ammonium in combination are often more efficacious than any one of these salts by itself. They may be given, well diluted, in a large dose once a day, or from five to ten grains may be repeated three times a day after meals. When the former method is employed, mercurial treatment by inunction or by hypodermic injection should be continued, but in the latter case perchloride of mercury in suitable dose may be combined with the iodide mixture. The chief drawback to this combination is that it is liable to give rise to pain in the bowels and troublesome diarrhœa, but these unpleasant results are often completely overcome by adding one or two minims of nepenthe to each dose. This treatment by mercury and the iodides, which is undoubtedly our most important means of combating syphilis of the eyes, must, if it is to be successful, be steadily continued for at least two years.

In conclusion, I have to thank Dr. Haswell Wilson for demonstrating the Wassermann reaction in case four, and my house surgeon, Dr. Walter Kiep, for the careful notes he made of all the patients.

